# Supplementary material for: The mRNA methyltransferase Mettl3 modulates cytokine mRNA stability and limits functional responses in mast cells
Source: Nat Commun. 2023 Jun 29;14:3862. doi: 10.1038/s41467-023-39614-y (PMC10310798; doi:10.1038/s41467-023-39614-y)
Supplement: Supplementary file 1 — Supplementary Information [file 41467_2023_39614_MOESM1_ESM.pdf]

The mRNA methyltransferase Mettl3 modulates cytokine mRNA stability and limits functional responses in mast cells

Leoni C., Bataclan M. *et al.*

Supplementary Information

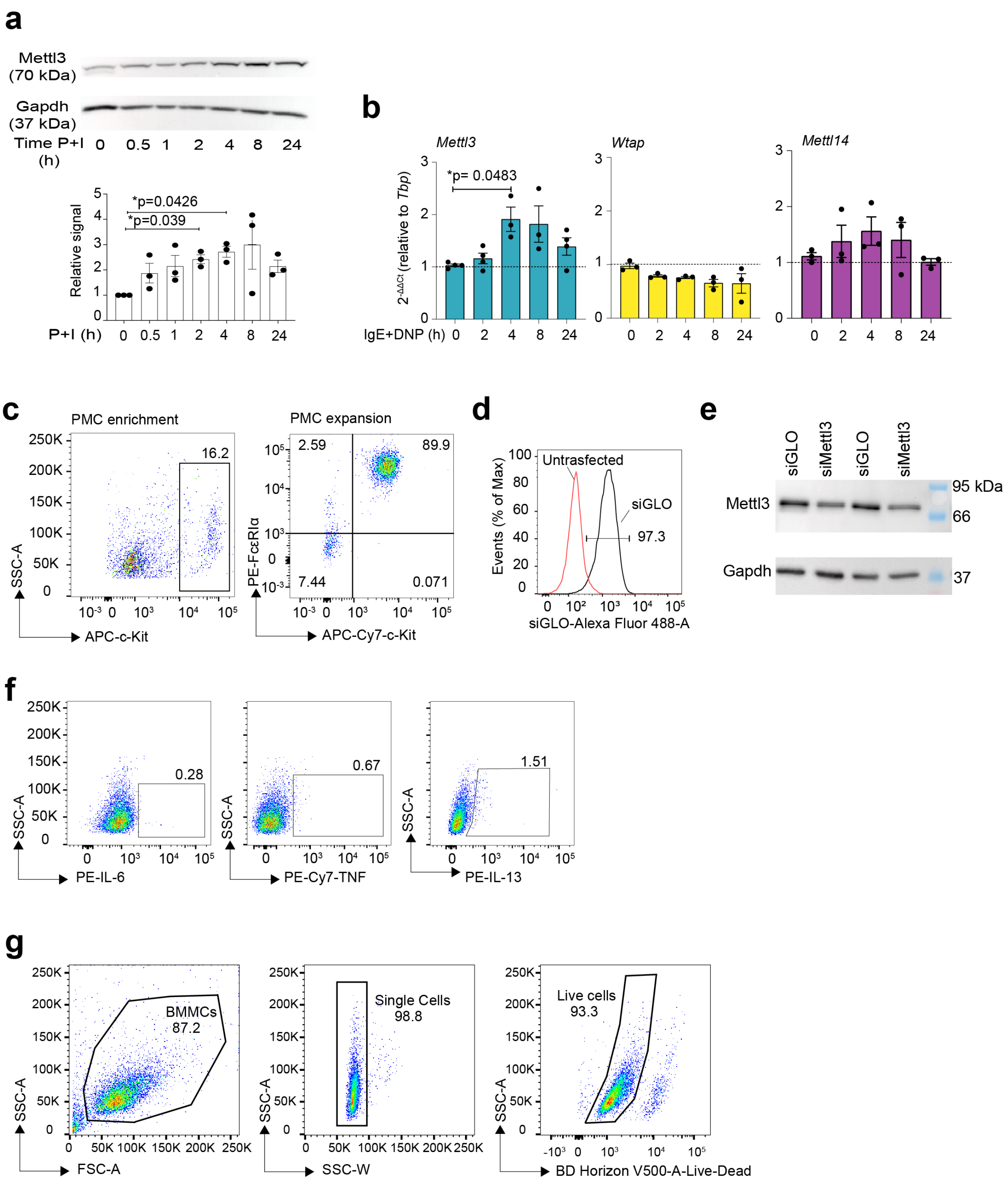

Supplementary Figure 1. Mettl3 expression is induced upon acute stimulation of mast cells. a) Mast cells were stimulated with PMA and ionomycin for the indicated times and expression of Mettl3 was analyzed by western blot. One representative blot is shown, with the quantification of N=3 independent experiments. Mean ± SEM. One-way ANOVA. **b)** Mast cells were stimulated with IgE and antigen for the indicated times. RT-qPCR was performed to measure the expression of the indicated transcripts. N=3 independent experiments. Mean ± SEM. One-way ANOVA. **c)** Analysis of *ex-vivo*-derived PMCs with

gating used for enrichment (left) and with their phenotype after one week of *in vitro* expansion with SCF. **d)** Mast cells were transfected with a control, fluorescent oligonucleotide to assess the efficiency of transfection. Representative of N>10 independent experiments. **e)** Cells were transfected with siRNAs against Mettl3 or control, followed, 48 h later, by western blot analysis. Two representative experiments are shown. **f)** Intracellular staining for the indicated cytokines in resting, unstimulated mast cells. **g)** Gating scheme for mast cell analyses. Source data are provided as a Source Data File.

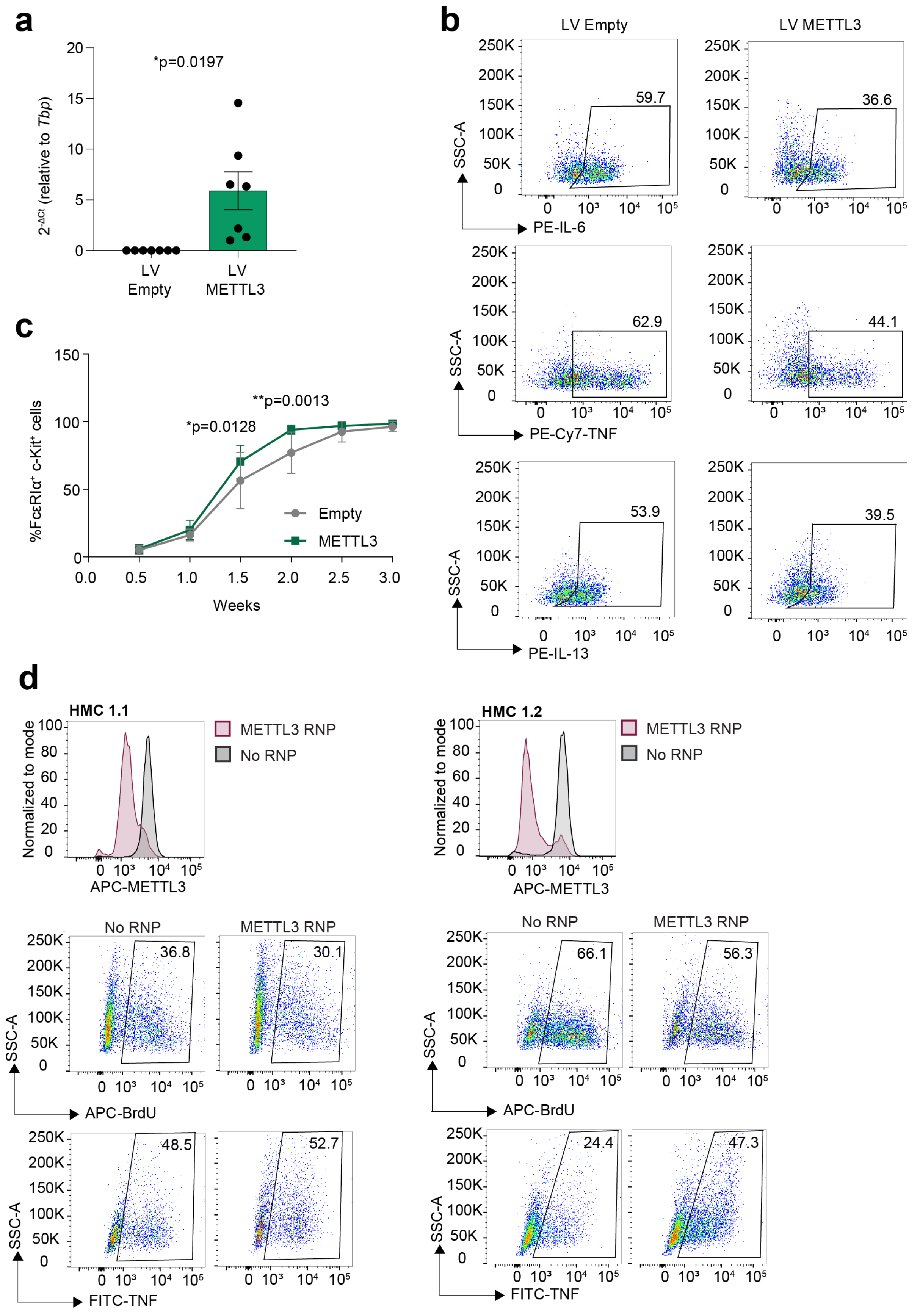
a
*p=0.0197
$2^{-\Delta Ct}$ (relative to *Tbp*)
LV Empty
LV METTL3
b
LV Empty
LV METTL3
59.7
36.6
SSC-A
PE-IL-6
62.9
44.1
PE-Cy7-TNF
53.9
39.5
PE-IL-13
c
**p=0.0013
*p=0.0128
%FcεRIα⁺ c-Kit⁺ cells
Empty
METTL3
Weeks
d
HMC 1.1
METTL3 RNP
No RNP
Normalized to mode
APC-METTL3
No RNP
METTL3 RNP
36.8
30.1
APC-BrdU
48.5
52.7
FITC-TNF
HMC 1.2
METTL3 RNP
No RNP
66.1
56.3
24.4
47.3

Supplementary Figure 2. Altered expression of human METTL3 affects mast cell differentiation and cytokine production. a) Expression of *METTL3* mRNA in mast cells transduced with a lentiviral vector to force METTL3 expression. N=7 independent experiments. Mean ± SEM. Paired t-test, two-tailed. **b)** Intracellular cytokine staining in cells transduced as in a) and stimulated with IgE+antigen complexes. **c)** Lineage-negative bone marrow precursors were transduced 24 h after isolation with the indicated vectors, followed by sorting for GFP expression and measurement of the indicated markers to assess mast cell differentiation over time. N=9 independent experiments. Mean ± SEM. Two-way ANOVA. **d)** Two human mast cell lines (HMC 1.1 and 1.2) were transfected with RNPs to ablate human METTL3. Intracellular staining for METTL3 revealed nearly complete ablation (top). Proliferation in these cells was measured by BrdU incorporation (middle). Cells were also stimulated with PMA and ionomycin followed by intracellular staining to measure TNF expression (bottom). Representative of N=2 independent experiments, each with two independent cell lines. Source data are provided as a Source Data File.

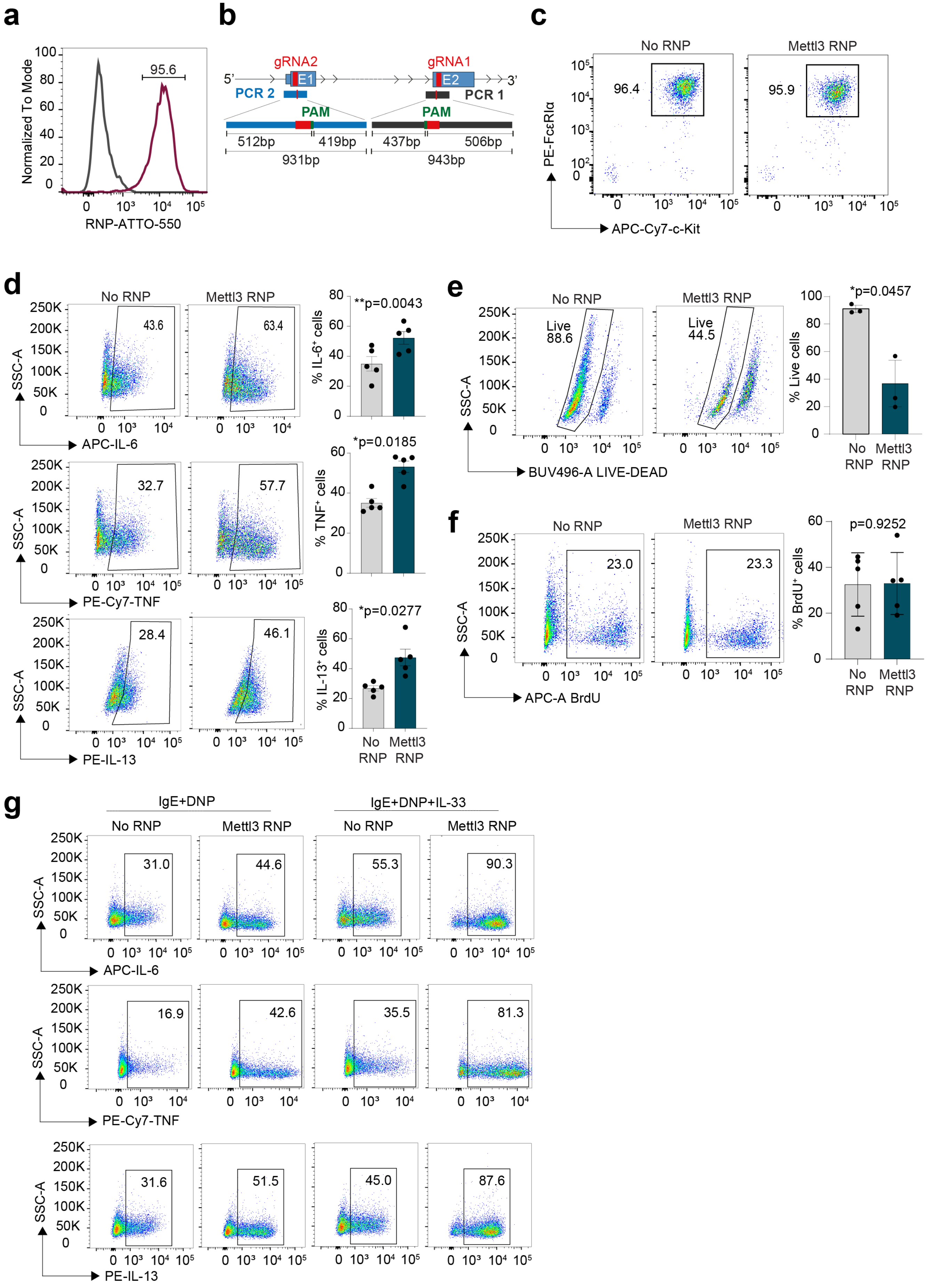
a
Normalized To Mode
95.6
RNP-ATTO-550
b
gRNA2
gRNA1
5'
E1
E2
3'
PCR 2
PCR 1
PAM
PAM
512bp
419bp
437bp
506bp
931bp
943bp
c
No RNP
Mettl3 RNP
PE-FcεRIα
96.4
95.9
APC-Cy7-c-Kit
d
No RNP
Mettl3 RNP
43.6
63.4
SSC-A
APC-IL-6
**p=0.0043
% IL-6+ cells
32.7
57.7
PE-Cy7-TNF
*p=0.0185
% TNF+ cells
28.4
46.1
PE-IL-13
*p=0.0277
% IL-13+ cells
No RNP
Mettl3 RNP
e
No RNP
Mettl3 RNP
Live 88.6
Live 44.5
BUV496-A LIVE-DEAD
*p=0.0457
% Live cells
f
No RNP
Mettl3 RNP
23.0
23.3
APC-A BrdU
p=0.9252
% BrdU+ cells
g
IgE+DNP
IgE+DNP+IL-33
No RNP
Mettl3 RNP
No RNP
Mettl3 RNP
31.0
44.6
55.3
90.3
APC-IL-6
16.9
42.6
35.5
81.3
PE-Cy7-TNF
31.6
51.5
45.0
87.6
PE-IL-13

Supplementary Figure 3. Deletion of Mettl3 affects mast cell physiology in a variety of experimental conditions. a) One representative FACS plot showing the efficiency of mast cell transfection using RNPs containing a fluorescent tracrRNA. **b)** Schematic representation of the *Mettl3* locus with indicated the locations of the gRNAs used in this study, the PCRs used for T7 Endonuclease I assay and the expected sizes of DNA digestion in this assay. **c)** One representative FACS plot showing normal c-Kit and FcεRIα expression by mast cells transfected with Mettl3 RNPs. **d-f)** Cytokine expression (d), viability (e) and proliferation (f) of BMMCs differentiated in the presence of IL-3 + SCF and depleted of Mettl3 by CRISPR-Cas9. Mean ± SD. Paired t-test, two-tailed. **g)** Cytokine expression in cells lacking Mettl3 and stimulated with IgE+antigen and 0.1 ng/ml IL-33 for 4h. Representative of N=2 independent experiments. Source data are provided as a Source Data File.

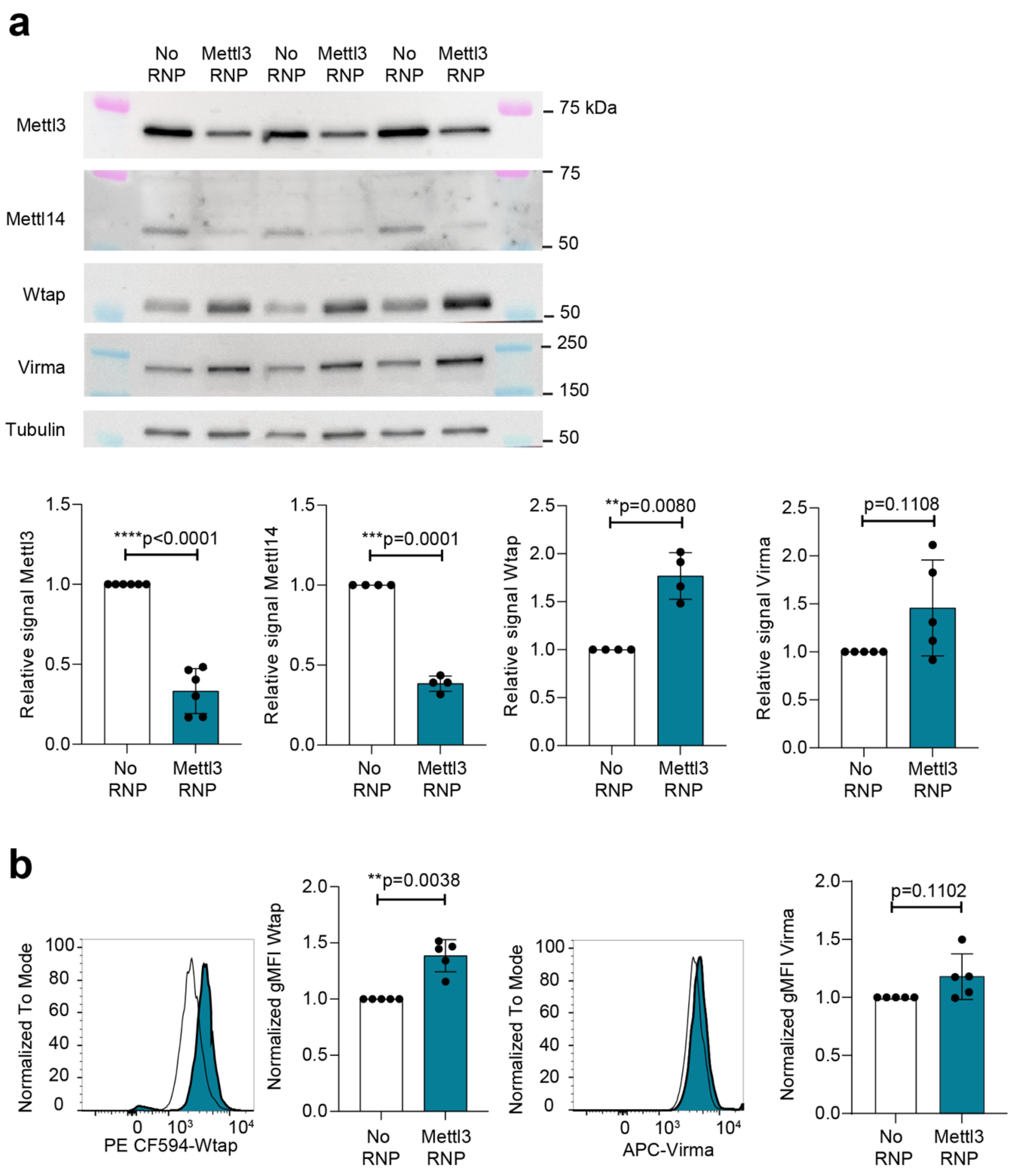

Supplementary Figure 4. Deletion of Mettl3 affects the expression of Wtap and Mettl14. a) Mast cells were transfected with Mettl3 RNPs, followed by western blot of the indicated components of the m^{6}A methyltransferase complex. **b)** In cells as in a), the expression of Wtap and Virma was measured by intracellular staining. Each dot represents one experiment. N=5 independent experiments (ratio compared to control samples). Mean ± SD. Paired t-test, two-tailed. Source data are provided as a Source Data File.

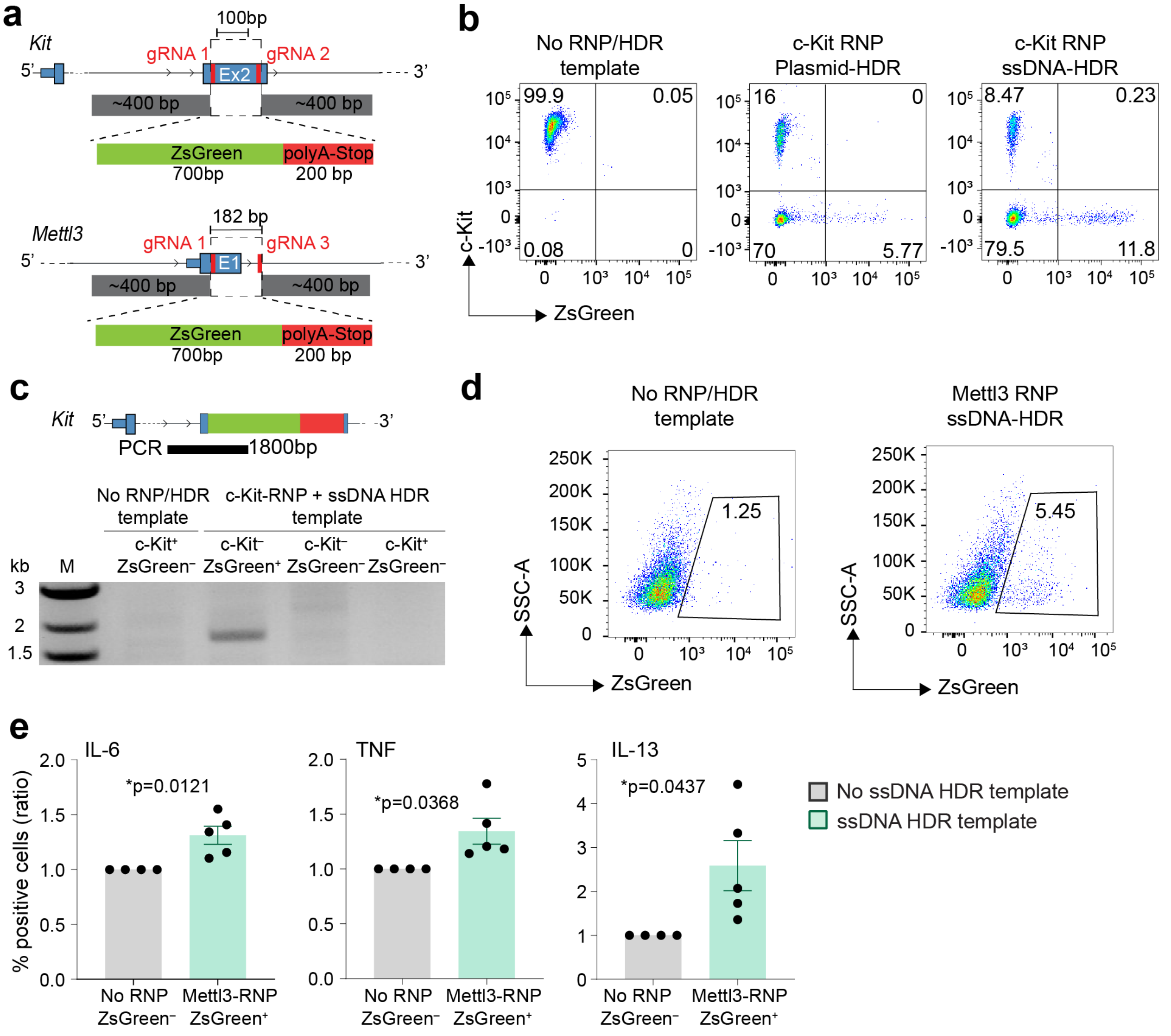

Supplementary Figure 5. Homology-directed repair for gene replacement in mast cells. a) Schematic representation of the *Kit* and *Mettl3* gene loci, indicating the positions of the gRNAs used (exon 2 for the *Kit* gene and exon 1 for *Mettl3*), the homology arms, and the ZsGreen-polyA-Stop cassette used to insert the ZsGreen reporter in the endogenous locus while at the same time disrupting expression of the gene of interest. **b)** Mast cells were transfected with RNPs containing gRNAs against the *Kit* gene, together with the HDR template as in a). As donor template, either the entire plasmid or ssDNA encompassing the ZsGreen-polyA-Stop cassette and homology arms were used. One representative experiment of N=2 (plasmid) or N=4 (ssDNA) is shown. **c)** Top, schematic representation of a targeted *Kit* locus, with indicated the PCR used to detect the insertion in the locus. Bottom, one representative result of correct targeting (N=4). M=marker. **d)** Mast cells were transfected with RNPs containing gRNAs against the *Mettl3* gene, together with the ssDNA HDR template. ZsGreen expression was measure 72 h after transfection. Representative of N=4 experiments. **e)** Intracellular cytokine staining in cells transfected as in d). N=4-5 independent experiments (ratio compared to control samples). Mean ± SEM. Unpaired t-test, two-tailed. Source data are provided as a Source Data File.

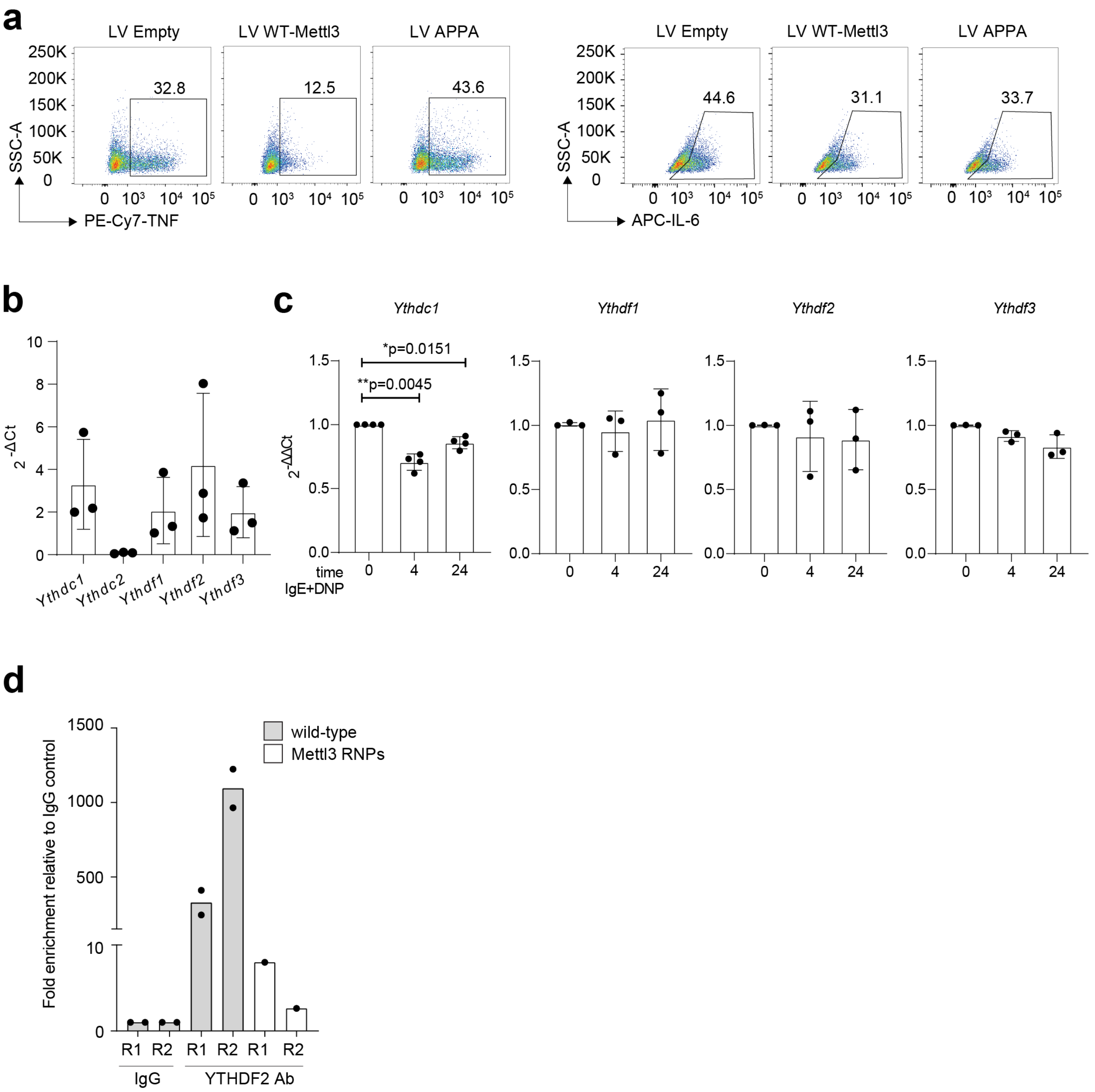

Supplementary Figure 6. Expression of m^{6}A readers in mast cells. a) Representative FACS plots related to Figure 8c. **b)** Expression of the indicated transcripts was measured in resting mast cells by RT-qPCR. N=3 independent experiments. Mean ± SD. **c)** Expression of the indicated transcripts was measured by RT-qPCR in mast cells stimulated for 4 h or 24 h. N=3-4 independent experiments. Mean ± SD. One-way ANOVA. **d)** RNA-immunoprecipation (RIP) using an anti-YTHDF2 antibody was performed on stimulated mast cells. After washing and RNA extraction, RT-qPCR was performed using two sets of primers located in two different regions within the *Il13* 3'UTR. Deletion of Mettl3 by CRISPR-Cas9 reduced the RIP signal, suggesting that YTHDF2 recognizes methylated regions within the 3'UTR of the *Il13* transcript. RIP-2 (R2) primers are located exactly on the m^{6}A peak identified by m^{6}A-CLIP-seq. Data were normalized on the values of the IgG controls. Mean ± SEM. Representative of N=2 independent experiments. Source data are provided as a Source Data File.

Supplementary Table 1. Antibodies used in this study.

Antibody	Catalog number
anti-CD117 (c-Kit)-APC	Biolegend, 105812
anti-CD117 (c-Kit)-APC/Cy7	Biolegend, 105825
anti-FcεRIα-PE	Biolegend, 134307
anti-CD11b (Mac-1)-Pacific Blue	Biolegend, 101223
anti-Ly-6G (Gr-1)-PE-Cyanine7	eBioscience, 25-5931-81
IgE–anti-DNP	Sigma, D8406
anti-BrdU-Alexa Fluor 647	BD Biosciences, 560209
anti-IL-6-PE	Biolegend, 504503
anti-IL-6-APC	Biolegend, 504507
anti-TNF-α-PE/Cy7	Biolegend, 506323
anti-IL-13-PE	eBioscience, 12-7133-41
anti-human-TNF-FITC	Biolegend, 502906
anti-METTL3 (clone EPR18810)	Abcam, ab195352
anti-WTAP (clone 4A10G9)	Proteintech, 60188-1-Ig
anti-METTL14 (clone D8K8W)	Cell Signaling Technology, 51104S
anti-Virma (clone D4N8B)	Cell Signaling Technology, 88358S
anti-YTHDF2 (clone EPR20318)	Abcam, ab220163
anti-GAPDH	Sigma, G9545
anti-beta-tubulin (clone 1D4A4)	Proteintech, 66240-1-Ig
anti-rabbit IgG (H+L) Alexa Fluor 647	Sigma, SAB4600393
anti-mouse IgG (H+L) Alexa Fluor 647	ThermoFisher Scientific, A21236
anti-mouse IgG (H+L) Alexa Fluor 594	ThermoFisher Scientific, A11005
anti-rabbit whole IgG (whole molecule)-HRP	Sigma, A0545
anti-mouse IgG (H+L)-HRP	SouthernBiotech, Cat nr. 1031-05
Normal rabbit IgG	Cell Signaling Technology, 2729S

Supplementary Table 2. Oligonucleotides and siRNAs used in this study.

	Primer FW 5’→3’	Primer RV 5’→3’
**CRISPR/ T7**		
crRNA_1 *Mettl3* T7 endonuc.	CTGAGATCCCAGTTTTGATTATTG A	GGTCACTGTAGTCAAATCCTGTTCT
crRNA_2 *Mettl3* T7 endonuc.	CAAGGACCAGAATATACAATCAA CC	CTCTGGGGATAGATATACAGAAGCA
Kit HDR integration PCR	TGAGTGCCTGTGTTCATTCAC	CCTTGTACACGGTGTCGAACT
**Sybr green RT-qPCR**		
*Mettl3*_sybr	GACACGTGGAGCTCTATCCAG	CAGTAGGCACGGGACTATCAC
*Wtap*_sybr	AGCAGCAACAGCAGGAGTCT	CTGCTGAACTTGCTTGAGGTACT
*Mettl14*_sybr	GGAACGCAGAGCTTAAATCC	GCTATTAACTCATCCTTTAGTCTGATG
*Csf2*_sybr	GAAGTCGTCTCTAACGAGTTCTCC	TATGTCTGGTAGTAGCTGGCTGTC
*Il13*_sybr	GCATGGTATGGAGTGTGGAC	ATTGGAGATGTTGGTCAGGG
*Il2*_sybr	CCAGGATGCTCACCTTCAAAT	ATGAAATTCTCAGCATCTTCCAAT
*Il3*_sybr	AAACTGATGATGAAGGACCCTCT	GTATCTGTCCTCAGGATCCACTTC
*Tnf*_sybr	CTTCTGTCTACTGAACTTCGGG	CAGGCTTGTCACTCGAATTTTG
*Ythdc1*_sybr	TTTTCAGGAGTTCGCCGAG	GTAAGGATGGTGTGGAGGTTG
*Ythdc2*_sybr	GGAGAGCAGCATGGTTTACTTGG	GGCTCTTCTCCCTTCCTATCTC
*Ythdf1*_sybr	TCATTATGAGAAGCGCCAGG	AGACAGCACCAAGCATACAG
*Ythdf2*_sybr	ACTATGAGAAACGCCAAGAGG	TTAGGATAAGGAGATGCAACCG
*Ythdf3*_sybr	TGCACATTATGAAAAGCGTCAAG	GGCATTTCCAGAGTCTACATC
*Tbp*_sybr	CTGGAATTGTACCGCAGCTT	ATGATGACTGCAGCAAATCG
**RIP-RT-qPCR**		
RIP *Il13* 3’UTR #1	TGGAAGAATGGCCTGTTACAC	TCACCATCTTTATTTCCGGTTT
RIP *Il13* 3’UTR #2	GCATCTCAGCTGTGGACTCATTTT CCTTTC	TGCCAGGCTGAGACCCTGAGCACTA

**siRNAs**		
Mettl3 siRNA	GGGATATTCACATGGAGCTACCGTATT	TACGGTAGCTCCATGTGAATATCCCTT
**DRACH mutagenesis**		
3'UTR_*Il13* site 1	TGAGGAGAGCGCTTCCCTGGGCATCTCAGC	GCTGAGATGCCCAGGGAAGCGCTCTCCTCA
3'UTR_*Il13* site 2	CCCTGGGCATCTCAGCTGTGGTACCATTTTCCTTTCTCACATCAA	TTGATGTGAGAAAGGAAAATGGTACCACAGCTGAGATGCCCAGGG
3'UTR_*Il13* site 3	ATTTTCCTTTCTCACATAGCACTAAGCTTGGGAGAGGCAG	CTGCCTCTCCCAAGCTTAGTGCTATGTGAGAAAGGAAAAT
