# Supplementary material for: The mRNA methyltransferase Mettl3 modulates cytokine mRNA stability and limits functional responses in mast cells
Source: Nat Commun. 2023 Jun 29;14:3862. doi: 10.1038/s41467-023-39614-y (PMC10310798; doi:10.1038/s41467-023-39614-y)
Supplement: Supplementary file 3 — Description of Additional Supplementary Files [file 41467_2023_39614_MOESM3_ESM.pdf]

Description of Additional Supplementary Files

File name: Supplementary Data 1
Description: RNA sequencing results (Figure 5a) with raw data, differential expression and gene ontology terms.

File name: Supplementary Data 2
Description: miCLIP-seq results, mast cells unstimulated and stimulated with PMA and ionomycin for 2 h (Figure 5).

File name: Supplementary Data 3
Description: Raw Nanostring counts (Figure 7).

File name: Supplementary Data 4
Description: Sequences and information related to HDR templates (Figure 6).
