# Supplementary material for: The mRNA methyltransferase Mettl3 modulates cytokine mRNA stability and limits functional responses in mast cells
Source: Nat Commun. 2023 Jun 29;14:3862. doi: 10.1038/s41467-023-39614-y (PMC10310798; doi:10.1038/s41467-023-39614-y)
Supplement: Supplementary file 8 — Reporting Summary [file 41467_2023_39614_MOESM8_ESM.pdf]

nature portfolio

Corresponding author(s): Silvia Monticelli
Last updated by author(s): Jun 9, 2023

Reporting Summary

Nature Portfolio wishes to improve the reproducibility of the work that we publish. This form provides structure for consistency and transparency in reporting. For further information on Nature Portfolio policies, see our Editorial Policies and the Editorial Policy Checklist.

Statistics

For all statistical analyses, confirm that the following items are present in the figure legend, table legend, main text, or Methods section.

n/a	Confirmed	
☐	☒	The exact sample size (n) for each experimental group/condition, given as a discrete number and unit of measurement
☐	☒	A statement on whether measurements were taken from distinct samples or whether the same sample was measured repeatedly
☐	☒	The statistical test(s) used AND whether they are one- or two-sided<br>*Only common tests should be described solely by name; describe more complex techniques in the Methods section.*
☒	☐	A description of all covariates tested
☐	☒	A description of any assumptions or corrections, such as tests of normality and adjustment for multiple comparisons
☐	☒	A full description of the statistical parameters including central tendency (e.g. means) or other basic estimates (e.g. regression coefficient) AND variation (e.g. standard deviation) or associated estimates of uncertainty (e.g. confidence intervals)
☐	☒	For null hypothesis testing, the test statistic (e.g. F, t, r) with confidence intervals, effect sizes, degrees of freedom and P value noted<br>*Give P values as exact values whenever suitable.*
☒	☐	For Bayesian analysis, information on the choice of priors and Markov chain Monte Carlo settings
☒	☐	For hierarchical and complex designs, identification of the appropriate level for tests and full reporting of outcomes
☒	☐	Estimates of effect sizes (e.g. Cohen's d, Pearson's r), indicating how they were calculated

Our web collection on statistics for biologists contains articles on many of the points above.

Software and code

Policy information about availability of computer code

Data collection	Flow cytometry data were collected on a FACS Symphony A5 or Fortessa (BD Biosciences).<br>qPCR data were collected an ABI 7900HT Fast Real-Time PCR system (Applied Biosystems) or a QuantStudio 3 Real-Time PCR System (ThermoFisher Scientific).
Data analysis	Flow cytometry data were analyzed by FlowJo version 10 (BD Bioscience).<br>Statistical analysis was performed with Prism version 9 (GraphPad).<br>Analysis of inflammatory transcript expression was performed using the nSolver Advanced Analysis Software v4.0 (NanoString Technologies).<br>Analysis of RNA-seq data was performed using R version 4.2.1 and the Omics Playground web-based platform.<br>Data visualization was performed with RStudio version 4.1 and IGV 2.16.0.<br>Protein band intensity was quantified using the ImageJ software version 1.53e.

For manuscripts utilizing custom algorithms or software that are central to the research but not yet described in published literature, software must be made available to editors and reviewers. We strongly encourage code deposition in a community repository (e.g. GitHub). See the Nature Portfolio guidelines for submitting code & software for further information.

Data

Policy information about availability of data

All manuscripts must include a data availability statement. This statement should provide the following information, where applicable:

- Accession codes, unique identifiers, or web links for publicly available datasets
- A description of any restrictions on data availability
- For clinical datasets or third party data, please ensure that the statement adheres to our policy

All data supporting the findings of this study are available within the paper and its supplementary information files. Datasets pertaining to sequencing and Nanostring profiling are deposited as a Super Series in GEO with accession number GSE228615.

Human research participants

Policy information about studies involving human research participants and Sex and Gender in Research.

Reporting on sex and gender	Not applicable
Population characteristics	Not applicable
Recruitment	Not applicable
Ethics oversight	Not applicable

Note that full information on the approval of the study protocol must also be provided in the manuscript.

Field-specific reporting

Please select the one below that is the best fit for your research. If you are not sure, read the appropriate sections before making your selection.

☒ Life sciences ☐ Behavioural & social sciences ☐ Ecological, evolutionary & environmental sciences

For a reference copy of the document with all sections, see nature.com/documents/nr-reporting-summary-flat.pdf

Life sciences study design

All studies must disclose on these points even when the disclosure is negative.

Sample size	No sample size calculation was performed. No statistical methods were used to pre-determine sample sizes, which was determined by a balance of technical feasibility and statistical power. At least 3 biological replicates were analyzed in each experiment. In vivo experiments were performed at least twice, each time with at least 3 biological replicates.
Data exclusions	No data exclusion of technically valid data was performed.
Replication	At least 3 biological replicates were analyzed in each experiment. In vivo experiments were performed at least twice, each time with at least 3 biological replicates. All attempts at replication, in the absence of any particular technical issue, were successful.
Randomization	Allocation of individual mice or cell batch to each experimental group was always random.
Blinding	In vivo experiments were performed in blind (the researcher physically performing the experiment was unaware of the identity of the samples, that were coded by a second researcher, until the end of the experiment). Blinding was not relevant (and therefore not performed) for other experiments, but experiments were for the most part repeated independently by two researchers to ensure reproducibility.

Reporting for specific materials, systems and methods

We require information from authors about some types of materials, experimental systems and methods used in many studies. Here, indicate whether each material, system or method listed is relevant to your study. If you are not sure if a list item applies to your research, read the appropriate section before selecting a response.

Materials & experimental systems

n/a	Involved in the study	
☐	☒	Antibodies
☐	☒	Eukaryotic cell lines
☒	☐	Palaeontology and archaeology
☐	☒	Animals and other organisms
☒	☐	Clinical data
☒	☐	Dual use research of concern

Methods

n/a	Involved in the study	
☒	☐	ChIP-seq
☐	☒	Flow cytometry
☒	☐	MRI-based neuroimaging

Antibodies

Antibodies used

anti-CD117 (c-Kit)-APC, Biolegend, Cat.nr. 105812
anti-CD117 (c-Kit)-APC/Cy7, Biolegend, Cat. nr. 105825
anti-FcεRIα-PE, Biolegend, Cat.nr. 134307
anti-CD11b (Mac-1)-Pacific Blue, Biolegend, Cat. nr. 101223
anti-Ly-6G (Gr-1)-PE-Cyanine7, eBioscience, Cat. nr. 25-5931-81
IgE–anti-DNP, Sigma, Cat. nr. D8406
anti-BrdU-Alexa Fluor 647, BD Biosciences, Cat. nr. 560209
anti-IL-6-PE, Biolegend, Cat. nr. 504503
anti-IL-6-APC, Biolegend, Cat. nr. 504507
anti-TNF-α-PE/Cy7, Biolegend, Cat. nr. 506323
anti-IL-13-PE, eBioscience, Cat. nr. 12-7133-41
anti-METTL3 antibody (clone EPR18810), Abcam, Cat. nr. ab195352
anti-WTAP antibody (clone 4A10G9), Proteintech, Cat. nr. 60188-1
anti-METTL14 antibody (clone D8K8W), Cell Signaling Technology, Cat. nr. 51104S
anti-Virma antibody (clone D4N8B), Cell Signaling Technology, Cat. nr. 88358S
anti-YTHDF2 antibody (clone EPR20318), Abcam, Cat. nr. ab220163
anti-GAPDH antibody, Sigma, Cat. nr. G9545
anti-beta-tubulin antibody, Proteintech, Cat. nr. 66240-1-Ig
anti-rabbit IgG (H+L) Alexa Fluor 647, Sigma, Cat. nr. SAB4600393
anti-mouse IgG (H+L) Alexa Fluor 647, ThermoFisher Scientific, Cat. nr. A21236
anti-mouse IgG (H+L) Alexa Fluor 594, ThermoFisher Scientific, Cat. nr. A11005
anti-rabbit whole IgG-HRP antibody, Sigma, Cat. nr. A0545
anti-mouse IgG (H+L)-HRP antibody, SouthernBiotech, Cat nr. 1031-05
anti-mouse IgG (light-chain specific)-HRP antibody, Jackson ImmunoResearch, Cat. nr. 115-035-174
Normal rabbit IgG, Cell Signaling Technology, Cat. nr. 2729S

Validation

We used commonly used antibodies from the literature. Validation was performed by the vendor or as reported by the Protein Atlas (https://www.proteinatlas.org) and for primary antibodies was reported as follows:
anti-CD117 verified reactivity against mouse and quality control tested for flow cytometry
anti-FcεRIα verified reactivity against mouse and quality control tested for flow cytometry
anti-CD11b verified reactivity against mouse and quality control tested for flow cytometry
anti-Ly-6G verified reactivity against mouse and quality control tested for flow cytometry
IgE–anti-DNP tested in allergic reactions in vivo, flow cytometry, immunocytochemistry, mast cell degranulation
anti-BrdU tested for bioimaging
anti-IL-6 verified reactivity against mouse and quality control tested for intracellular staining with flow cytometry
anti-TNF verified reactivity against mouse and quality control tested for intracellular staining with flow cytometry
anti-IL-13 verified reactivity against mouse and quality control tested for intracellular staining with flow cytometry
anti-METTL3 reacts with human, mouse and rat, tested for western blot, immunohistochemistry, immunofluorescence, immunoprecipitation, knock-out validated
anti-WTAP recognizes human, mouse and rat, tested for western blot, immunohistochemistry, immunofluorescence
anti-METTL14 recognizes human, mouse, rat, monkey. Approved for western blot.
anti-Virma recognizes human, mouse, rat, monkey. Approved for western blot and immunoprecipitation.
anti-YTHDF2 reacts with human, mouse and rat, tested for western blot, immunoprecipitation, knock-out validated
anti-GAPDH recognizes human, mouse, and rat GAPDH, tested for western blot
anti-beta-tubulin recognizes human, mouse, rat and 3 more species, tested for western blot, immunohistochemistry, immunofluorescence, immunoprecipitation, ELISA

Eukaryotic cell lines

Policy information about cell lines and Sex and Gender in Research

Cell line source(s)

HEK293T cells were obtained from ATCC.
HMC-1.1 and 1.2 human mast cell lines were kindly provided by Joseph Butterfield.

Authentication

HEK293T cells were not authenticated.
HMC-1.1 and 1.2 human mast cell lines were not authenticated.

March 2021

Mycoplasma contamination	Cell lines tested negative for mycoplasma. Primary cell cultures have a very limited life-span, and are newly generated for each experiment, so they are not tested.
Commonly misidentified lines (See ICLAC register)	No cell line that have been reported as misidentified has been used.

Animals and other research organisms

Policy information about studies involving animals; ARRIVE guidelines recommended for reporting animal research, and Sex and Gender in Research

Laboratory animals	6-8 week old C57Bl/6 mice (males and females) were used for bone marrow and peritoneal cell cultures. KitWsh/W-sh mice lacking mast cells were obtained from The Jackson Laboratory. The mice were housed in specific pathogen-free conditions, with 12h dark/light cycle, 20-24C temperature and 50-65% humidity.
Wild animals	No wild animals were used in this study.
Reporting on sex	Both male and female animals were used in this study.
Field-collected samples	No field-collected samples were used in this study.
Ethics oversight	All animal studies were performed in accordance with Swiss Federal Veterinary Office guidelines and approved by the Cantonal animal experimentation committee, Dipartimento della Sanità e della Socialità Cantone Ticino (authorization number TI10/19).

Note that full information on the approval of the study protocol must also be provided in the manuscript.

Flow Cytometry

Plots

Confirm that:

☒ The axis labels state the marker and fluorochrome used (e.g. CD4-FITC).

☒ The axis scales are clearly visible. Include numbers along axes only for bottom left plot of group (a 'group' is an analysis of identical markers).

☒ All plots are contour plots with outliers or pseudocolor plots.

☒ A numerical value for number of cells or percentage (with statistics) is provided.

Methodology

Sample preparation	Bone marrow was flushed out of femurs and tibias and plated in tissue culture flasks with complete medium. Adherent cells were removed in the following days by changing the flasks.<br>Peritoneal cells from intraperitoneal washings were plated in tissue culture flasks with complete medium. Adherent cells were removed in the following days by changing the flasks.
Instrument	FACS Symphony A5 or Fortessa (BD Biosciences) was used for data collection.<br>FACS Symphony S6 (BD Biosciences) was used for cell sorting.
Software	Flow cytometry data were analyzed using FlowJo v10.6.0
Cell population abundance	Very variable depending on the experimental conditions. At least 10'000 events/ samples are usually recorded.
Gating strategy	Cells were gated for the mast cell population (SSC-A/ FSC-A), single cells (SSC-A/SSC-W) and living cells using LIVE/DEAD-Fixable Aqua Dead Cell Stain or LIVE/DEAD-Fixable Blue Dead Cell Stain Kit (ThermoFisher Scientific).

☒ Tick this box to confirm that a figure exemplifying the gating strategy is provided in the Supplementary Information.
